# How much are we willing to do for the ones we love – impact on caregivers of patients suffering from periprosthetic joint infections: a qualitative study

**DOI:** 10.5194/jbji-11-105-2026

**Published:** 2026-02-10

**Authors:** Franz-Joseph Dally, Franziska Prüßner, Frederic Bludau, Sascha Gravius, Ali Darwich, Marcel Betsch

**Affiliations:** 1 Department of Orthopaedic and Trauma Surgery, University Medical Centre Mannheim, University of Heidelberg, Theodor-Kutzer-Ufer 1–3, 68167 Mannheim, Germany; 2 Department of Trauma and Orthopaedic Surgery, University Erlangen, Maximiliansplatz 2, 91054 Erlangen, Germany

## Abstract

**Introduction**: We aimed to identify and explore the psychological and physical strains that caregivers of patients suffering from a periprosthetic joint infection (PJI) experience. **Methods**: Twenty-four qualitative semi-structured interviews with people giving care to patients suffering from a PJI were conducted by a single trained interviewer. The interviews were used to identify physical and emotional challenges that caregivers were confronted with. **Results**: The psychological and physical strains caregivers reported were wide-ranging and included suffering from stress and anxieties, and feelings of hopelessness and helplessness while having to function all the time to the point where, subjectively, caregivers felt as if they gave themselves up. Caregivers were suffering from mental and physical exhaustion as mainly family members and close ones provided the caregiving. Oftentimes an elaborate team effort approach was needed to lighten the caregiving burden. **Conclusions**: This study shows that caregivers are willing to go above and beyond for their loved ones, while exhausting and exceeding their individual resources. Caregivers of PJI patients deal with major issues. Specifically, we identified (1) conflicts regarding the hospital stay, (2) novel personal challenges, (3) nursing, (4) emotional and psychological consequences, and (5) effects on the relationship. Our research shows that there is a profound need for support during and after hospitalisation, and caring for a PJI patient seems surprisingly similar to caring for a cancer patient. We recommend including the caregivers of PJI patients, and how to better support them, into the PJI treatment guidelines.

## 1 Introduction

A PJI can be devastating and has long-lasting effects on patients (Mallon et al., 2018; Moore et al., 2015; Dally et al., 2025). As reported, numbers of total joint arthroplasties are expected to rise over the next decades (Hegde et al., 2023); accordingly, numbers of revision surgeries and PJIs are also expected to rise. While the direct effects on PJI patients have been studied (Dally et al., 2025; Mallon et al., 2018), to date, the effects on caregivers, family members, and partners of PJI patients have not been studied. To study an individual experience, it can be sensible to conduct a qualitative study as qualitative research allows the participants to elaborate on their experiences, attitudes, and feelings during a certain time (Tenny et al., 2023). Qualitative studies are widely accepted in the psychology research field (Braun and Clarke, 2006) and have been adapted into other research areas where they help to gain further insights into clinical topics such as caregiving (Ringborg et al., 2022; Mallon et al., 2018; Tenny et al., 2023; Cleland, 2017; LeSeure and Chongkham-Ang, 2015).

We conducted this study to investigate the impact of caring for a PJI patient. As literature on caregivers of PJI patients was not available, we compared our results to widely accepted burdens of cancer caregivers as this is the most researched area of caregiving (LeSeure and Chongkham-Ang, 2015).

## 2 Methods

From January to August 2023, a total of 24 caregivers, family members, or partners of 22 PJI patients were interviewed (semi-open questions) for this qualitative study.

We identified the PJI patients and their caregivers using the respective ICD-10 codes in our electronic patient database. The diagnosis had been made using the MSIS guidelines (Parvizi et al., 2018). An invitation email was sent to the patients, inviting their partners or caregivers to participate in a telephone interview. Recruitment was halted when no new themes emerged over two consecutive interviews, to ensure an appropriate sample size as is standard procedure in qualitative studies and which has previously been described as data saturation (Clarke and Braun, 2021; Mallon et al., 2018; Guest et al., 2006). This point of saturation reflects the number of interviews after which no new information regarding the research question “what is the impact on caregivers of patients suffering from a PJI” is found.

Ethics approval was obtained before the start of the study by the local ethics board. All participating caregivers and patients gave their written consent. The study protocol was created in accordance with the Consolidated Criteria for Reporting Qualitative (COREQ) Studies (Tong et al., 2007).

The questions were developed by the study guide and multiple different experts in the field of psychology, pain medicine, orthopaedics, and periprosthetic infection research, and reviewed by at least two other authors and agreed upon after two pilot interviews to test usability.

### 2.1 Qualitative data analysis

Initially, we analysed the data using general inductive analysis. At least three team members (Franz-Joseph Dally, Franziska Prüßner, and Marcel Betsch) conducted a thematic analysis of the caregivers' interview transcripts using the inductive process previously described by Braun and Clarke (2006).

The authors made themselves familiar with the data by reading and re-reading the interviews. Individually at first, the authors reviewed the transcripts and coded the data. In team meetings, more reviewing and interpreting of the data led to identifying patterns, which in turn are referred to as themes (Malterud, 2001; Tenny et al., 2023; Braun and Clarke, 2006). The coding process was aided by commercial software (MAXQDA Software; Verbi Software, Berlin, Germany) (Vignato et al., 2022). During the coding process via text comprehension, answers or issues were graded in a numerical order according to their importance to the interviewees and therefore categorised according to their relevance or power, helping to establish themes. Agreeing to these themes took multiple team meetings to ensure a dependable and trustworthy process (Clarke and Braun, 2021).

### 2.2 Quantitative data

We gathered quantitative data such as number of children, number of family members, and length of caregiving, and the associated bacteria found causative for the PJI.

Regarding the PJI patients, we gathered information on kind of primary arthroplasty, patients' side of surgery, BMI, number of surgeries, pathogens detected, and kind of infection (acute vs chronic).

The statistical analysis was performed with Microsoft Excel^®^ (Microsoft Corp. Excel 2019).

Quantitative parameters were presented as mean values with ranges and standard deviations.

### 2.3 Socio-demographic information

We gathered information such as sex, age, occupation, relation to the PJI patient, and information on living arrangements.

Regarding the PJI patients, we gathered information on age, sex, living arrangements, and number of relatives.

The statistical analysis was performed with Microsoft Excel^®^ (Microsoft Corp. Excel 2019).

## 3 Results

### 3.1 Study population

As depicted in Table 1, the caregivers of the PJI patients were predominantly female (79 %, 19/24). The largest group was spouses (46 %, 11/24). A total of 42 % were children (10/24), and 12 % (3/24) were parents. Mean age of the caregivers was 57.3 years (standard deviation – SD – of 15.2 years). The duration of caregiving ranged from 2 weeks to 7 years. A total of 75 % (18/24) caregivers were living with the PJI patients, one of whom moved in with the patient. A total of 50 % (12/24) of caregivers were employed full time, 33 % (8/24) were retired, 8 % (2/24) left the job to take care of the PJI patient, and 8 % (2/24) were already at home being a “stay-at-home spouse”.

**Table 1:** Key characteristics of the caregiver study population.

Parameters	N (%)
Age	mean 57.3 years (range: 25–82)
Sex	
Male	6 (17)
Female	18 (83)
Relationship	
Spouses	11 (46)
Children	10 (42)
Parents	3 (12)
Living arrangements	
Living with the PJI^*^ patient	18 (75)
Living separate from the PJI^*^ patient	6 (25)
Working status	
Employed full time	12 (50)
Retired	8 (33)
Left job to care for PJI^*^ patient	2 (8)
Already “stay-at-home spouse”	2 (8)

^*^ PJI: periprosthetic joint infection.

We held 24 semi-open interviews with 24 caregivers of 22 PJI patients (the questions used can be found in Table S1 in the Supplement). Of two patients, we separately interviewed two caregivers.

As depicted in Table 2, 73 % (16/22) of the PJI patients were suffering from a PJI of the hip, and 17 % (6/22) patients were suffering from a PJI of the knee. Mean age of the PJI patients was 70.7 years (SD: 11.7 years), with a mean BMI of 28.6 kg m^−2^ (SD: 7.3 kg m^−2^). A total of 68 % (15/22) of patients were suffering from a chronic PJI, and 32 % (7/22) of patients suffered from an acute PJI according to the recent guidelines for PJI diagnosis and treatment (Parvizi et al., 2018; McNally et al., 2021). A total of 32 % (7/22) were male PJI patients, and 68 % (15/22) were female PJI patients. Five patients suffering from a PJI of the hip joint received a spacer at one point during their treatment protocol. The number of revision surgeries ranged from one to 22 surgeries for one PJI patient. The PJI patient with 22 surgeries suffered from a PJI of the hip and the last known outcome was discharge with a revision hip endoprosthesis.

**Table 2:** Key characteristics of the PJI patients.

Parameters	N (%)
Age	mean 70.7 years (range, 55–83)
Sex	
Male	7 (32)
Female	15 (68)
BMI^1^	mean 28.6 kg m^−2^ (range: 18.1–46.4)
Periprosthetic joint infections	
Acute	7 (32)
Chronic	15 (68)
Primary prosthesis implanted	
Total knee arthroplasty	6 (17)
Total hip arthroplasty	16 (73)
Revision protocol	
One-stage revision surgery (DAIR^2^)	5 (23)
Multiple-stage revision surgeries	17 (77)
Infection type	
Mono-microbial	5 (23)
Poly-microbial	17 (77)
Number of surgeries	Ranging from one to 22 surgeries
Spacer	5 (23)
Last known outcome	
Retained prosthesis or revision prosthesis	13 (59)
Girdlestone	5 (23)
Arthrodesis	2 (9)
Amputation	2 (9)

^1^ BMI: body mass index. ^2^ DAIR: debridement, antibiotics and implant retention.

At one point of this study, the largest group of 59% (13/22) of the PJI patients had been discharged with a prosthesis. A total of 23 % (5/22) of patients had a girdlestone situation, 9 % (2/22) of patients had received a septic arthrodesis (resection of infected area and bone-to-bone arthrodesis by fixateur externe or implantation of arthrodesis prosthesis), 4 % (1/22) of patients had received a hip disarticulation for a PJI of the hip that was deemed life-threatening, and 9 % (2/22) of patients had received an above-knee amputation for a PJI of the knee that was deemed uncontrollable.

### 3.2 Qualitative research results

**Figure 1:**
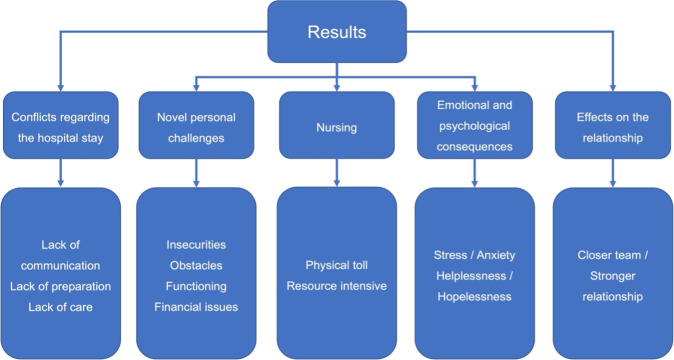
Schematic representation of the five main themes and subthemes regarding caregiving of PJI patients.

The qualitative interview study revealed what caregivers were going through by caring for a PJI patient. This study shows wide-ranging effects – both positive and negative. After careful analysis of the semi-open qualitative interviews, we identified five main themes, as depicted in Fig. 1. Notable quotes are added; a more extensive list can be found in Table S1.

#### 3.2.1 Conflicts regarding the hospital stay

Many caregivers felt left alone with their questions about the course of treatment and the meaning of a PJI in general. They felt that communication from doctors and nurses was lacking openness and straight-forwardness. Others reported that reaching doctors seemed impossible. As a result, a sense of real frustration arose.

… essential for a relative or close one is a transparent communication, and we know there is a lot of pressure on doctors in hospitals and university clinics and they might be in the operating room all day, it is still paramount to take the time for the relatives and the people caring for the PJI patients and answering all of their questions and to manage their fears and anxieties … (Caregiver 1 – CG 1).

Participants claimed being left with insufficient training or information on caring for a patient and feeling overwhelmed with the situation at home. Some mentioned how nothing, not even a job in nursing, but especially not the hospital staff, could prepare them for the challenges.

… nobody prepared us for the tasks we were facing, one day my father was just discharged and arrived at home … (CG 2).

Some PJI patients developed bed sores, which influenced the quality of life and prolonged the course of treatment of the PJI patient by making the process to a re-implantation of a prosthesis more difficult. Bed sores presented an unknown and novel issue to deal with after discharge.

… We were hoping he could receive another hip prosthesis, but because of the bed sores he had developed on his feet, they had to heal before another surgery (CG 3).

#### 3.2.2 Novel personal challenges

All caregivers faced a plethora of new obstacles, many regarding nursing, medical issues, immobilisation and wound secretion, or wound care. Being medically untrained and unprofessional, many had to overcome obstacles suddenly and adapt on the fly, which strained personal physical and psychological resources and challenged everyone concerned.

… I really had to do everything and it was tough (CG 4).

When elaborating on the challenges, many reported being under constant pressure to function – functioning for themselves and for their loved ones suffering from the PJI, all while functioning in their regular job and private life. One participant felt like she gave herself up because she was needed all the time, every day.

… It really was a 24 h job. Basically, the entire time next to my regular job was taken up with the task of caring for my father (CG 5).

Participants reported on diminishing financial funds from losing one income earner or from paying for a nursing home or nursing service. Two participants left their jobs to care for their loved ones; others had to take over administrative and financial tasks previously handled by their partner.

… That really scares me. I have existential fears. We were trying to accumulate some wealth and all of a sudden, my husband couldn't earn income anymore (CG 6).

#### 3.2.3 Nursing

The participants struggled from having to take care of their loved ones in a matter in which they had never taken care of them before, causing physical and mental exhaustion. One participant mentioned that it felt as if there was no way out and being utterly exhausted.

… I was suffering from exhaustion. You feel as if you just keep working all day and it never ends; there is not one calm second in a day (CG 7).

Personal resources are massively strained by trying to maintain a regular life, working a regular job, and maintaining their own family activities (household, cooking, shopping) and family life. Some participants needed help from the entire family to meet the required demands.

… I have two sons and both supported us, and they still support us to this day … (CG 8).

#### 3.2.4 Emotional and psychological consequences

The PJI and the ordeal of caring for their loved ones caused wide-ranging consequences at the emotional and psychological level, ranging from stress and anxiety to being worried all the time while being fully emerged in the care for a person suffering from the PJI.

… All of it was such a challenge and the lack of sleep and the worrying. It was the worst time (CG 9).

Participants mentioned feeling hopeful before and after surgeries but in some cases being disappointed over and over again, to the point of feeling helpless and hopeless.

… Every time before a surgery there was this hope of everything turning out well this time, only to find out that there was another infection. We always had this hope and were disappointed after a while (CG 10).

#### 3.2.5 Effects on the relationship

Some participants felt that the emotional closeness improved. Some reported connecting through vulnerability and overcoming challenging times together. They pointed out that on many levels, these positive effects helped them to keep going.

… because of this whole ordeal we definitely got closer … (CG 11).

## 4 Discussion

A total of 32 % (7/22) were male PJI patients and 68 % (15/22) were female PJI patients. The proportions tilt towards female PJI patients but is close to our previous study with a large PJI cohort (169 patients; 49.7 % female and 50.3 % male), which showed an almost even distribution between the sexes (Darwich et al., 2025). While we cannot fully explain why female PJI patients dominated in this cohort, we can only assume that female patients were more likely to agree to be included in this study. The female PJI cohort was not focus of this study, but the caregivers and the female dominance of the caregivers is glaring and comparable to other studies (Sharma et al., 2016).

The mean BMI in this cohort was 28.6 kg m^−2^ (range: 18.1–46.4). This is comparable to other studies which have shown that a higher BMI is associated with a higher risk of PJI (Zhong et al., 2020). The impact of the elevated BMI of the PJI patients had no impact of note on the caregivers.

While caregiving, or the burden of caregiving in the medical field regarding differing patient groups, has been extensively researched, the burden of caregiving to PJI patients is yet to be studied (Bedaso et al., 2022; Cadet et al., 2016; Mystakidou et al., 2007; Nijboer et al., 1998; Northouse et al., 2012; Palacio et al., 2020; Ringborg et al., 2022; Sklenarova et al., 2015; Snowden et al., 2018; Tranberg et al., 2021; Clarke and Braun, 2021; LeSeure and Chongkham-Ang, 2015). Interestingly, our study reveals a previously unknown similarity to caring for a cancer patient. As has been described in cancer patient care, caregiving becomes a full-time job; supportive activities include household tasks, emotional support, managing money, and cancer patient care can have both a positive and a negative impact on the caregivers (LeSeure and Chongkham-Ang, 2015). This is supported by our study on caring for a PJI patient as our participants reported being harshly impacted by the taxing physical demands. They reported being overwhelmed by the challenge of balancing the needs of their loved one suffering from the PJI with their own personal lives and professional careers. Some moved in with the person they cared for, some left their jobs, many reported financial issues. On the other hand, a great portion of our participants experienced a positive satisfactory effect and a newfound closeness on an emotional level, for instance, by overcoming daily challenges together.

Our participants overwhelmingly reported how caregiving for a PJI patient is physically taxing and may be so for an extended period, depending on the course of the PJI and its treatment. This compares to the previously reported physical impact when caring for cancer patients. Interestingly, many our participants mentioned this aspect in their interviews, although almost 1/4 of PJI patients received a DAIR procedure and generally would not be thought of as needing much care. Throughout our study cohort, there was a sense of being overwhelmed both physically and psychologically and feeling left alone with the tasks, challenges, and requirements a PJI patient needs. These descriptions again best align with the impact of caring for and living with a cancer patient (LeSeure and Chongkham-Ang, 2015).

The very high demand for personal care, help, and nursing left the participants wishing for more support, nursing help, and practical advice.

Some participants reported that they had to adjust their entire lives to the new situation. Some changed or quit their jobs, some felt they neglected the rest of their family and their social life. One participant described the feeling “as losing myself”. To summarise, caregivers of PJI patients suffer from emotional and psychological consequences such as stress, anxiety, hopelessness, and helplessness, and some even broke down completely. These experiences compare well to those reported by cancer patient caregivers (Ringborg et al., 2022; LeSeure and Chongkham-Ang, 2015). As cancer caregivers often do, our participants reported prioritising the PJI patient's life over their own and worrying about the future almost more than the patients themselves (Ringborg et al., 2022; Tranberg et al., 2021; Northouse et al., 2012).

While it has already been reported how devastating a PJI can be for patients and how patients need a greater support system, we were able to show for the first time that this need translates to their caregivers as well (Mallon et al., 2018). PJI patients in the Mallon et al. study cohort reported how they did not feel taken seriously when they first presented with their symptoms and how communication was problematic when speaking to healthcare professionals (doctors, nurses, etc.) (Mallon et al., 2018). This was in line with our study where participants complained about the communication with and from healthcare professionals. Consequently, we advise healthcare professionals to take and make enough time for their patients and loved ones. An improved communication can improve the caregiver's perception, can help to keep a positive mindset, and can strengthen the patient–doctor–caregiver relationship. Furthermore, healthcare professionals can enhance the positive aspect of a caregiver's perception when regularly providing information, emotional support, and effective medical treatment (LeSeure and Chongkham-Ang, 2015).

As Sklenarova et al. showed for cancer patients, there are many unmet needs of caregivers, causing depression, anxiety, and other issues (Sklenarova et al., 2015). Our study shows that this is also true for the caregivers of PJI patients, showing there is a need for further research and support on all levels for caregivers of PJI patients. They face the same emotional burden as somebody caring for an elderly person, a demented person, a stroke victim, or a cancer patient (LeSeure and Chongkham-Ang, 2015).

The results of Ringborg et al.'s study on caregivers for patients with oesophageal cancer showed that caregivers particularly struggle with being solely responsible for the well-being of the patients. To them it feels like nothing will be the same again and as if their sense of self is lost (Ringborg et al., 2022). In the present study, we were able to confirm these findings. Our participants reported struggling with being responsible for everything at home, developing anxiety relating to their and the patient's future, and feeling that their own life has taken a back seat to the needs of the PJI patient. There are a multitude of qualitative studies that have studied the effect that cancer has on caregivers, but our study is the first to report these effects on caregivers of PJI patients (Ringborg et al., 2022; Northouse et al., 2012; Tranberg et al., 2021).

For caregivers of cancer patients, there are holistic models established to improve their lives and situations (Cadet et al., 2016); for caregivers of PJI patients, these have not been formulated. Furthermore, while there are methods and entire support systems being developed and financed to support caregivers of cancer patients, and psychological consultations for cancer patients and their next of kin are readily available, these have not been adapted to the caregivers of PJI patients.

Our research shows that throughout the heterogenous group of PJI patients, all the caregivers suffer in one way or another. This is a new insight into the socio-economic burden of a PJI, which extends far beyond the patient. Therefore, we recommend offering psychological consultations for caregivers and improving the support caregivers receive regarding communication, preparation, nursing, physical therapy, financial advice, and outpatient care.

There are guidelines and recommendations on how to diagnose and treat a PJI, published by specialists in their respective fields – mainly orthopaedic surgeons and infectiologists or microbiologists (McNally et al., 2021; Parvizi et al., 2018). However, these guidelines so far do not include any information or methods on how to inform, support, or involve the caregivers in the process of PJI treatment (McNally et al., 2021; Parvizi et al., 2018). We advocate including the family members and caregivers into the guidelines of PJI treatment as they shoulder a great burden.

To summarise, caregivers are willing to go to great lengths and endure many hardships for the PJI patients, but they suffer physically, privately, individually, as a family, and psychologically very similar to the way caregivers do for cancer patients. On the other side, caregivers of PJI patients do find positive feedback and impact in their care and relationship with their loved ones.

## 5 Limitations

The heterogeneous aspect of the PJI patient cohort ( 3/4 of PJI patients having undergone multi-revisions surgeries, 1/4 having undergone a DAIR procedure) can be argued as a limitation. Since the kind of operative procedure undertaken was not focus of this study, we view this limitation as weak at best. Our caregiver cohort compares well to other caregiver groups, as stated above.

## 6 Conclusion

PJI patient care has both positive and negative impacts on caregivers. Through this study, we identified five main themes that greatly impacted our study cohort: (1) conflicts regarding the hospital stay, (2) novel personal challenges, (3) nursing, (4) emotional and psychological consequences, and (5) effects on the relationship.

To conclude, caregivers of PJI patients shoulder a relevant burden physically and emotionally, which seems very similar to caregivers for cancer patients. While further research is warranted, an inclusion in established treatment algorithms for PJI should be considered, focusing on the aspects revealed in this study.

## Supplement

10.5194/jbji-11-105-2026-supplement The supplement related to this article is available online at https://doi.org/10.5194/jbji-11-105-2026-supplement.

## Data Availability

The data presented in this study are available on request from the corresponding author.
