# Supplementary material for: How much are we willing to do for the ones we love – impact on caregivers of patients suffering from periprosthetic joint infections: a qualitative study
Source: J Bone Jt Infect. 2026 Feb 10;11(1):105–12. doi: 10.5194/jbji-11-105-2026 (PMC12917602; doi:10.5194/jbji-11-105-2026)
Supplement: The supplement related to this article is available online at https://doi.org/10.5194/jbji-11-105-2026-supplement. [file jbji-11-105-2026-supplement.pdf]

Supplement of J. Bone Joint Infect., 11, 105–112, 2026
https://doi.org/10.5194/jbji-11-105-2026-supplement
© Author(s) 2026. CC BY 4.0 License.

JBJI
Journal of Bone and Joint Infection
Open Access

Supplement of

How much are we willing to do for the ones we love – impact on caregivers of patients suffering from periprosthetic joint infections: a qualitative study

Franz-Joseph Dally et al.

Correspondence to: Franz-Joseph Dally (franz.dally@umm.de)

The copyright of individual parts of the supplement might differ from the article licence.

Supplement S1 JBJI

Questions used during the interview process:

1. Please elaborate on the general situation regarding the hospital stay of your close one suffering from a PJI.

a. What has been most memorable for you regarding the treatment in the hospital?

b. What changed for you personally after the diagnosis

2. Please elaborate on the relationship between you and your loved one (e.g. spouse, daughter, friend)

a. How long have you been in a caregiving/care-partner relationship and what kind of care did you or do you provide to your family member

b. Did something change regarding your relationship?

c. Do you openly communicate your fears and worries with each other?

3. Please elaborate on the physical relationship to your loved one

a. Has the physicality changed after the diagnosis?

b. Were you able to care for your loved one on your own or did you need help/support

c. Were you able to handle the medial parts such as injections, wound dressing and such?

4. Please elaborate on the relationship within your family regarding the caregiving to the loved one suffering from a PJI

a. Did you have help/support from your family members, or did you re-ceive professional help from an ambulatory care service?

b. Did you receive emotional support in any way?

c. What was the most challenging for you?

5. Please elaborate on how the caregiving has affected your relationship to friends, neighbors and family members

a. What are the most rewarding parts of being a caregiver? What are the most challenging aspects of being a caregiver?

Quotes used to develop subthemes and main themes

Table S1. Quotations regarding the key theme "Effects on the relationship"

**Key theme**	**Effects on the relationship**
Closer Team / stronger bond	› „…over the course of these last three years **we became such a close team.** …" › „…because of this whole ordeal we **definitely got closer**. I would go as far as saying other people would be jealous off our relationship. We realized how important the time together really is. …" › „…after all these years, our **relationship actually got stronger** because of the infection. We care even more about each other now. …" › „…**Our relationship has changed to the better**, we are more open with each other and tell each other what we need, **are more honest with each other.** ..."

Table S2. Quotations regarding the key theme: "Emotional and psychological consequences"

**Key theme**	**Emotional and psychological consequences**
Stress/Anxiety	› „…during that time, I was under **constant stress**. It seems as if you aren't living, you just keep taking care of one task after another that don't seem to end. …" › „…All of it was such a challenge and the lack of sleep and the worrying. **It was the worst time.** …" › „…My mother was **overwhelmed with the whole situation**, and we were fearing for my father's life. …"
Helplessness / Hopelessness	› „…Every time before a surgery there was this **hope of everything turning out well** this time, only to find out that there was another infection. We always had this hope and **were disappointed after a while.** …" › „…The issue is that as a family you have this expectation and hope that from receiving another prosthesis everything will turn to the better and that the

	quality of life will improve. **None of us could foresee that this would be the result.** …"<br>› „…You just **feel exposed, because you yourself can't do anything**, you have to trust the doctors and end up feeling helpless. …"

Table S3. Quotations regarding the key theme "Nursing":

Key theme	Nursing
Physical toll	› „…I was **suffering from exhaustion**. You feel as if you just keep working all day and it never ends, there is not one calm second in a day. …"<br>› „…**I put my own life second**, there was nothing else, you are just there for this person (with the PJI). You take days off work to accompany him for doctor's appointments. It felt like self-sacrificing at times. …"<br>› „…**I had to take care of everything** and therefore learn everything anew. Every task that needed to be done. …"
Resource intensive	› „… **we had to do it**, a nursing home wouldn't even take her at her age of 53. …"<br>› „…**I have two sons and both supported us**, and they still support us to this day. …"<br>› „…**we take turns** with the caregiving and **we have developed a plan, a schedule** for my brothers and me. If I was doing this alone, I couldn't do it. …"

Table S4. Quotations regarding the key theme "Novel Personal Challenges":

Key theme	Novel Personal Challenges
Insecurities and Obstacles	› „…all of the sudden I was facing **so many obstacles**, I had to become the proxy, talk to the insurance, fill out forms. All these administrative tasks take up so much time and energy. ..."<br>› „… I really **had to do everything** and it was tough even though I work as an ICU nurse…"<br>› „…My father was discharged one day with all the medications and that was it. **We were left alone with this situation and had to learn how to do everything ourselves on the fly.** …"
Functioning	› „…It really was a **24h job**. Basically, the entire time next to my regular job was taken up with the task of caring for my father. …"<br>› „…Nobody asks how you are doing. People inquired about my father, because that is on their mind, the PJI and all the surgeries, but nobody asks: How are you doing? **You just have to function, period.** …"
Financial issues	› „…**That really scares me. I have existential fears**. We were trying to accumulate some wealth and all of a sudden, my husband couldn't earn income anymore. …"<br>› „…Obviously you could **put my husband in a home, but that is expensive** as well. A nursing service is not cheap either. These are **some issues that I keep worrying about.** …"

Table S5. Quotations regarding the key theme "Conflicts regarding the hospital stay"

Key theme	Conflicts regarding the hospital stay
Lack of communication	› „…the doctors rotated almost daily and seemingly no one passes on relevant information. We had the part of informing nurses and doctors and physical therapists, **but we are no experts.** …"<br>› „…**the communication was very difficult**; it seemed nurses and doctors didn't communicate sufficiently and the doctors didn't seem to communicate with each other either. …"<br>› „…essential for a relative or close one is **a transparent communication**, and we know there is a lot of pressure on doctors in hospitals and university clinics and they might be in the operating room all day, it is still paramount **to take the time** for the relatives and the people caring for the PJI patients and answering all of their questions and to manage their fears and anxieties. …"

Lack of preparation	› „…**nobody prepared us** for the tasks we were facing, one day my father was just discharged and arrived at home. …“<br>› „…there is always this **fear and uncertainty** when applying an injection. I mean I had seen it before in the hospital, but it is completely different when all of the sudden I had to do it. …”<br>› „…we knew the dressing had to be changed and renewed every other day, but then because of the infection it had to be done more often but we **had not prepared for this. ..."**
Lack of care	› „…my father had developed such a **horrible bed sore**. I was shown a photograph and I had never seen something like this, so I cried. It still effects his quality of life to this day. …“<br>› „…We were hoping he could receive another hip prosthesis, but **because of the bed sores** he had developed on his feet, they had to heal before another surgery. …”<br>› „… **The Situation in the hospital at times was very drastic**, I started to wonder who would take care of me when I got old. ..."

Inclusion and exclusion criteria:

Inclusion: any caregiver who cared for the PJI patients

Exclusion: caregiver younger than 18 years or unable to consent to this study

Definitions:
Multi-stage revision surgeries are all PJI patients that underwent implant removal, spacer implantation and at some point replantation or salvage surgery (e.g. fistula, amputation, arthrodesis)
One-stage revision surgeries in this study cohort exclusively refers to DAIR as we did not perform implant removal and replantation in one surgery.

Pathogens detected:

We included 5 patients with mono-microbial infections in our study. 17 patients suffered from poly-microbial infections. *Staphylococcus Epidermidis*, which was detected in 16/22 (72%) patients of our cohort was predominantly detected as depicted in **Table 3**, interestingly only in 2 of those cases it was detected alone, as a mono-microbial infection.
Given the nature of the qualitative study, we could not conclude if there is a significant correlation between type of infection (mono-microbial vs. poly-microbial) or type of pathogen identified and type of answer during the interviews.

Table S6. Identified pathogens

Pathogen	N (%)
Staphylococcus Epidermidis	16 (73)
Staphylococcus Aureus	8 (36)
Staphylococcus Capitis	2 (9)
Staphylococcus Hominis	2 (9)
Cutibacterium Acnes	3 (14)
Corynebacterium amycolatum	2 (9)
Enterococcus faecium	2 (9)
Enterococcus faecialis	2 (9)
Staphylococcus Haemolyticus	5 (23)
Staphylococcus caprae	2 (9)
Pseudomonas aeruginosa	3 (14)
Staphylococcus saccharolyticus	1 (5)

Finegoldia magna	1 (5)
Staphylococcus warneri	1 (5)
Pseudomonas oryzihabitans	1 (5)
Klebsiella aerogenes	1 (5)
Enterobacter cloacae spp.	1 (5)
Klebsiella pneumoniae	1 (5)
Acinetobacter Iwoffii	1 (5)
